# Salvage of failed free flaps used in head and neck reconstruction

**DOI:** 10.1186/1758-3284-1-33

**Published:** 2009-08-21

**Authors:** Daniel Novakovic, Rajan S Patel, David P Goldstein, Patrick J Gullane

**Affiliations:** 1 Department of Otolaryngology-Head and Neck Surgery, Princess Margaret Hospital, and University of Toronto, Toronto, Canada; 2 Department of Otolaryngology-Head and Neck Surgery, Auckland City Hospital, University of Auckland, Auckland, New Zealand

## Abstract

Free flap success rates are in excess of 95%. Vascular occlusion (thrombosis) remains the primary reason for flap loss, with venous thrombosis being more common than arterial occlusion. The majority of flap failures occur within the first 48 hours. With early recognition and intervention of flap compromise salvage is possible. Successful salvage rates range from 28% to over 90%. Rapid re-exploration in this clinical setting is crucial to maximise the chances of flap salvage. If salvage is not feasible or unsuccessful then non-surgical methods of salvage may be employed with some possibility of success. The purpose of this article is to review the causes of free flap failure and to highlight the available options for salvage.

## 

Free tissue transfer has become the gold standard in reconstruction of many Head and Neck surgical defects. With the development of new flaps, refinements in surgical technique, larger surgical volumes and technological advancements, free flap success rates in most high volume centers are in excess of 95% [1-6].

Free flap failure can lead to functional and cosmetic morbidity, as well as result in additional operative procedures, prolonged hospital stay and increased health care costs. Moreover, free flap failure with the development of oropharyngocutaneous fistula may increase the risk of lethal complications such as rupture of great vessels. All series report a certain incidence of flap failure. Early detection of flap compromise via careful monitoring and appropriate surgical revision can lead to significant improvements in overall success rates and is the subject of this review

## Causes of failure

Vascular occlusion (thrombosis) remains the primary reason for flap loss, with venous thrombosis being more common than arterial occlusion. The majority of flap failures occur within the first 48 hours. A number of authors have investigated the causes and timing of flap failure. In a series of 990 patients Kroll et al[6] reported that 50 cases (5.1%) developed pedicle thrombosis. Venous thrombosis was more than twice as common as arterial thrombosis and tended to develop later. Hidalgo et al[4] identified venous problems (35%) as the most common etiology of flap failure followed by arterial problems (28%), hematoma (26%) and recipient vessel problems (11%). Late flap failures (i.e. > 48 hours) were most often due to infection or mechanical stress around the anastomosis. In 756 cases Miyasaka et al[7] performed 22 explorations for vascular pedicle compromise, 17 (77%) of which were due to venous compromise and five due to an arterial problem. Most cases of venous compromise were identified within the first 25 hours following surgery. Brown et al[8] reviewed 427 free tissue transfers with 16% requiring return to the operating theatre within seven days for compromised flap or hematoma. Venous compromise (83%) was once again much higher than arterial compromise (8%).

There are many different causes for thrombosis or occlusion of the vascular pedicle. Technical errors with flap design and elevation, vessel suturing, tissue handling, and/or geometry of the pedicle may result in thrombosis. Extrinsic compression of the vascular pedicle by tight wound closure, tapes around the neck or wound hematoma may also compromise the flap by obstruction of venous outflow[9]. The use of interposition vein grafts has been shown to increase the risk of flap failure[10,11]. The recipient site and donor vessel choice may also be a factor in the development of flap failure. Although not consistently reported some authors have found prior radiotherapy at the recipient site to increase the risk of failure[11,12]. Choosing an appropriate donor vein and artery is important. In a review of 156 free flaps Chalian et al[2] found a significantly higher failure rate in flaps with venous anastomosis to the external jugular system compared with the internal jugular venous system. Flap success rates were 92% and 99% respectively. Ichinose et al[13] recommended the use of dual venous drainage (external and internal jugular systems) for the radial forearm free flap. They reported no venous failure in 405 consecutive cases. Significant medical co-morbidities, such as diabetes, hyper-coagulable disorders and alcohol withdrawal may result in an increased risk of flap failure[10,14,15].

## Salvage rates following free flap failure

With early recognition and intervention of flap compromise salvage is possible. Successful salvage rates range from 28% to over 90%[3,4,8,16,17]. The rates vary depending upon the etiology, timing of salvage and experience of the centre. In a review of 150 cases Hidalgo[9] suggested that attempted salvage of compromised flaps significantly increases flap survival rates and recommended an aggressive approach towards early exploration. In Brown's series[8] 73% of failed free flaps were successfully salvaged. Most of these were within 24 hours of initial operation and salvage rates were significantly higher for radial forearm than for composite flaps. In a multicenter survey Hirigoyen and Urken[3] found an initial failure rate of 6.7% with a salvage rate of 41% and an overall free flap success rate of 96%. Salvage rates were related to monthly case volume of free tissue transfers.

Salvage rates are higher when venous thrombosis is identified as the problem. In Nakatsuka's review[18] arterial thrombectomy was successful in only 15% of cases compared with a 60% salvage rate after venous thrombectomy. This may be related to the fact that venous compromise is easier to detect via traditional methods of monitoring[19]. Brown[8] concurred that arterial thrombosis was more difficult to detect due to lack of venous congestion but they did manage to salvage three of four flaps with pure arterial compromise; two of these being 72 hours after the initial operation. Hidalgo et al[4] in their series of 716 free flaps reported an eight percent re-exploration rate for vascular compromise with a successful salvage rate of 70%. Flap loss was much higher in buried flaps (7%) compared with non-buried flaps (2%) with a longer time to re-exploration in the buried group due to unreliable flap monitoring.

Salvage rates with late exploration are generally poor. Hyodo et al[20] reported that 4.1% of 513 cases of free flap reconstruction for head and neck defects were re-explored for postoperative thrombosis. Of these 21 cases 13 (62%) were due to venous thrombosis with a mean exploration time of 2.2 days after surgery. There was one case of arterial thrombosis at four days, with infection (4 cases) and anatomic variation or dissection error (3 cases) making up the remainder of the series. Successful salvage was possible in 33% (7/21) of flaps – all of these being due to venous thrombosis within the first 3 days of surgery. Mean re-exploration time for salvage cases was 1.3 days compared with 3.9 days for those not salvaged.

## Managing flap failure

The first step in managing free-flap failure is early recognition of a compromised flap. Clinical observation remains the simplest method of identifying vascular compromise[21]. Adjuncts such as pin-prick, temperature measurement and surface doppler are also used to aid in early recognition of problems. Buried flaps are more difficult to monitor clinically. An external skin monitor paddle may be used, otherwise monitoring relies on doppler signal, the loss of which should be a cause for immediate concern. An implantable doppler has also been demonstrated as an effective tool for monitoring flaps and potentially improving salvage rates[22,23]. Kind et al[22] suggested that a miniature doppler ultrasonic probe attached directly to the outflow vein of the flap may lead to a significant improvement in the salvage rates of free flaps. They identified 20 instances of vascular compromise in 147 free flaps using this technique with a salvage rate of 100%.

Upon suspicion of vascular compromise one should have a low threshold for return to the operating room for re-exploration[8]. Kubo et al[24] reviewed the management of the flap with venous compromise and suggested that surgical methods should be the first choice as it offers significantly higher salvage rates. Furthermore they felt that non-surgical procedures should only be used if surgical revision is not feasible or fails. With re-exploration initial attention should be directed at the vascular pedicle. Causes of extrinsic compression such as hematoma, pedicle kinking or misconfiguration are easily identifiable and potentially correctable. The internal jugular vein should also be examined for possible thrombosis[24] The arterial system should be examined under magnification for vascular spasm, for which topical Papaverine may be used. Arterial flow can be assessed by looking for pulsation of the distal pedicle or use of an intraoperative doppler. Milking of the venous system using microsurgical instruments may be used to assess venous outflow. Identification of thrombus should prompt opening the anastomosis and evacuation of the clot with heparinised saline irrigation or a Fogarty catheter prior to careful re-anastomosis[7]. Thrombolytic agents, such as streptokinase, urokinase or tissue plasminogen activator, can be used if a thrombus is identified, particularly in the venous system. Their use has been well documented as a means to salvage vascular insufficiency and theoretically prevent irreversible ischemic reperfusion injury or no-reflow phenomenon [19,24]. The venous anastomosis should be taken down prior to flushing the flap with any of these thrombolytic agents in order to avoid the systemic effects. Systemic antithrombotic therapy with intravenous heparin may be considered in select salvage cases of arterial or venous thrombosis where flow is re-established, particularly if thrombus formation rapidly occurs at the time of re-anastomosis. The drawback to intravenous heparin use is the potential for bleeding and hematoma formation. If thrombosis occurs at the time of re-anastomosis the initial recipient vein and/or artery may not be appropriate, in which case another should be chosen.

## Non-surgical management of the compromised flap

In select cases venous congestion can be managed with the application of leeches. Dabb[25] described several successful cases of venous congested flaps salvaged by leeches, suggesting that relief of congestion for four to ten days may allow enough time for neo-vascularisation. Neo-vascularization has been reported to occur as early as six days[26]. Leech therapy is primarily used in the management of venous congestion of flaps with a cutaneous portion used for external head and neck skin coverage. However, surgical re-exploration should be the first line of management of a compromised flap.

Partial flap loss may be managed with conservative treatment such as debridement and secondary healing. However, one must take into consideration the risk of conservative management, such as infection, or exposure of vital structures, as well as the type of flap, location and the indication for the flap when deciding on conservative management.

## Salvage reconstruction following Flap Loss

The success of salvage with re-exploration alone is related to the etiology and timing of flap failure and return to the operating room. The greatest chance of success will be in patients with a technical failure that is identified early with an immediate return to the operating room. The chance of surgical salvage is low after the first 48 hours. Hyodo et al[20] reported that flap salvage was impossible if thrombosis occurred more than three days after surgery. Late thrombosis was mainly due to fistula and local infection or mechanical stress around the anastamotic site, rather than technical failure. When salvage is not possible despite re-exploration and/or conservative management, a second flap usually needs to be performed. Salvage with a second flap may be performed in an immediate or delayed fashion, and may be either a second free flap or a regional flap. The timing and choice of flap used for salvage depends upon a number of factors including the original surgical defect, risk of wound infection, number of available flap options, and patient co-morbidities. Salvage reconstruction is technically very challenging since it occurs in a previously operated and often contaminated surgical bed and the ideal flap has already been utilized in the initial setting. Salvage reconstruction is particularly difficult in the head and neck, as critical structures, such as the great vessels and brain require coverage, infection, saliva and prior radiation or chemoradiation create a compromised wound bed, and patients are frequently malnourished with medical co-morbidities. Fearon[27] described a series of second attempts after failed initial free tissue transfer. Salvage surgery was more complicated due to factors such as depleted vessels for anastomosis but six of seven attempts were successful. The authors recommended careful consideration of the causes for initial free tissue transfer in order to correct any predisposing factors. Bozikov & Arnez[10] found that flap failure was 4.6 times more likely in a salvage setting with a success rate of only 53.3%.

Once the free flap used in the original reconstruction is deemed to be unsalvageable, salvage reconstruction should be performed as early as possible in order to avoid a severely compromised wound bed[16]. In cases where a severely compromised wound bed has developed initial conservative management with a delay in salvage reconstruction is recommended in order to increase the chance of success. The goals of salvage reconstruction are to select the simplest reconstructive option that will have the highest chance of survival but also is able to restore form, function and cosmesis. These goals are best achieved with a second free flap or a regional flap. In cases of a failed osteocutaneous flap for mandibular reconstruction a second osteocutaneous free flap should be used to restore both form and function. Patients who have had a failed total laryngopharyngectomy free flap reconstruction, a second free flap with either a tubed fasciocutaneous flap or enteric flap is recommended. Ideally, the reconstruction should be performed in an immediate fashion in order to allow patients to return to swallowing and speech rehabilitation as early as possible. However, in cases of infected wound beds conservative management with a pharyngostoma and esophagostoma may be required initially followed by secondary reconstruction once an ideal wound bed is achieved. In cases of flap failure following reconstruction of a partial pharyngectomy defect a second free flap or regional flap (pectoralis major or latissimus dorsi muscle) may be used. Pedicled flaps are particularly useful in the vessel-depleted neck. Failed intra-oral flaps can be salvaged with either a second fasciocutaneous free flap or regional flap. A second free flap is often the ideal reconstructive choice to provide the optimal functional result. Similarly, salvage reconstruction of large cutaneous defects can be performed with a pedicled latissimus dorsi or pectoralis major flap or a second cutaneous or musculocutaneous free flap. When a second free flap is used for reconstruction the flap success rate should be the most important factor in choosing the second flap, especially after loss of the first flap[16].

Availability of adequate vessels may be a significant problem in second free tissue transfer. When recipient veins are scarce for end-to-end anastomosis, the internal jugular vein may be used if available. Ueda[28] reviewed 948 free flaps and found that end to side venous anastomosis to the internal jugular vein was equivalent to end to end anastomosis and became their preferred technique of choice. Halvorson & Cordeiro[29] described a series of 320 free flaps utilising end to side anastomosis to internal jugular vein. They preferred this method of venous drainage due to size, constant anatomy, high patency rates and ready availability in most necks. In addition they felt there were less likely to be configuration problems associated with kinking even when the neck is turned. Similarly Yamamoto et al[30] preferred internal jugular vein end-to-side anastomosis citing advantages including potential for multiple anastomoses, potential beneficial respiratory venous pump effect and ability to overcome size discrepancy. Aycock et al[31] suggested that the thoracoacromial trunk vessels are a feasible option when first line vessels are not available. Jacobsen et al[32] described the use of cephalic vein transposition, transverse cervical vessels, thoracoacromial artery and Internal mammary vessels in a series of such cases. Use of these alternate vessels may require vein interposition grafting which has been associated with higher failure rates in other series[10,11].

## Conclusion

With adequate surgical experience and recent refinements in surgical technique free flap success rates should be in excess of 95%. Venous thrombosis is the most common cause of failure. Careful monitoring over the first 48 hours by experienced staff should allow for early identification of flap compromise. Rapid re-exploration in this clinical setting is crucial to maximise the chances of flap salvage. If salvage is not feasible or unsuccessful then non-surgical methods of salvage may be employed with some possibility of success. With flap loss there are a number if choices on the reconstructive ladder for defect correction. The use of a second free flap is feasible, but may be more technically demanding.

## Competing interests

The authors declare that they have no competing interests.

## Authors' contributions

1) All the authors (DN, RP, DG & PG) have made substantial contributions to conception and design, or acquisition of data, or analysis and interpretation of data;

2) All the authors have been involved in drafting the manuscript or revising it critically for important intellectual content; and

3) All the authors have read and given approval of the final manuscript version to be published. Each author has participated sufficiently in the work to take public responsibility for appropriate portions of the content.
